# Prediction of stress and drug craving ninety minutes in the future with passively collected GPS data

**DOI:** 10.1038/s41746-020-0234-6

**Published:** 2020-03-04

**Authors:** David H. Epstein, Matthew Tyburski, William J. Kowalczyk, Albert J. Burgess-Hull, Karran A. Phillips, Brenda L. Curtis, Kenzie L. Preston

**Affiliations:** Intramural Research Program, National Institute on Drug Abuse, 251 Bayview Blvd., Suite 200, Baltimore, MD 21224 USA

**Keywords:** Risk factors, Disease-free survival

## Abstract

Just-in-time adaptive interventions (JITAIs), typically smartphone apps, learn to deliver therapeutic content when users need it. The challenge is to “push” content at algorithmically chosen moments without making users trigger it with effortful input. We trained a randomForest algorithm to predict heroin craving, cocaine craving, or stress (reported via smartphone app 3x/day) 90 min into the future, using 16 weeks of field data from 189 outpatients being treated for opioid-use disorder. We used only one form of continuous input (along with person-level demographic data), collected passively: an indicator of environmental exposures along the past 5 h of movement, as assessed by GPS. Our models achieved excellent overall accuracy—as high as 0.93 by the end of 16 weeks of tailoring—but this was driven mostly by correct predictions of absence. For predictions of presence, “believability” (positive predictive value, PPV) usually peaked in the high 0.70s toward the end of the 16 weeks. When the prediction target was more rare, PPV was lower. Our findings complement those of other investigators who use machine learning with more broadly based “digital phenotyping” inputs to predict or detect mental and behavioral events. When target events are comparatively subtle, like stress or drug craving, accurate detection or prediction probably needs effortful input from users, not passive monitoring alone. We discuss ways in which accuracy is difficult to achieve or even assess, and warn that high overall accuracy (including high specificity) can mask the abundance of false alarms that low PPV reveals.

## Introduction

“Digital medicine” has many meanings, but one of the most exciting is the prospect of treating chronic disorders with just-in-time adaptive interventions (JITAIs).^1,2^ JITAIs, which currently exist in various stages of development and validation, are mobile treatments that learn to deliver therapeutic content exactly when patients need it. This is typically done via smartphone app. Among developers of JITAI apps, one major ambition is to “push” content to users at algorithmically chosen moments rather than relying on patients to “pull” the content themselves. This could be especially important for decisional events that are characterized by ambivalence, such as cravings and lapses in substance-use disorders (SUDs):^3^ at a watershed moment, an app-based interruption might help people make healthy decisions they would be less likely to make on their own.

The first hurdle to develop such a JITAI app is to give it the inputs it needs for prediction. Ideally, inputs would be collected by passive ambulatory monitoring, putting no burden on patients. Recent approaches to this problem have used digital phenotyping, a set of strategies that encompasses logging almost everything that can be sensed by a smartphone’s operating system.^4^ (We list many published examples in the “Discussion” section). Our research group, however, embarked on passive ambulatory monitoring in 2008, several years before smartphones approached their current levels of flexibility and ubiquity. We initially combined palmtop-computer ecological momentary assessment (EMA) with passive sensing of geolocation via stand-alone GPS loggers, a combination we call geographical momentary assessment (GMA).^5^ We developed GMA in the context of an NIH-wide initiative to develop methods for measuring environmental exposures;^6^ our main goal in that context was to acquire generalizable, population-level knowledge about the momentary dynamics of relationships between environment and behavior.^7^ Accordingly, we analyzed our GPS data not in terms of literal geospatial coordinates, but in terms of how places scored on observer-rated scales of psychologically relevant indices such as visible signs of poverty, violence, and drug activity.^8^

In our pilot GMA study, with 27 outpatients undergoing methadone maintenance for opioid-use disorder (OUD), we found that craving, stress, and mood were predicted by the past 5 h of exposure to visible signs of environmental disorder along a GPS-derived track.^5^ (The direction of the relationship was not always as we expected it to be, but, although that finding is heuristically important, it is not relevant for case-by-case prediction, so we do not discuss it further here). For those analyses, we used traditional inferential statistics—multilevel models that assess overall associations in whole samples and subgroups. The generalizable conclusions that can be drawn from inferential statistics do not explicitly quantify the proportion of people for whom they are not true^9^ or the number of moments at which they are not true. Explicit quantification of inaccuracy is the province of classification models, and the most accurate models are often so complex that they cannot contribute to generalizable knowledge.^10^ Investigators sometimes have to choose between explaining behavior (via the parsimony of inferential statistics) and correctly predicting it (via the multifactorial, interaction-laden models that typify machine learning).^11^ Accurate prediction is our goal in this paper.

For our machine-learning models, we collected GMA data from a new cohort of almost 200 outpatients with OUD, each assessed for up to 16 weeks during maintenance on methadone or buprenorphine at our clinic. All participants completed their data collection before we trained and tested the models; we used a high-performance server cluster (NIH Biowulf), accessed via desktop computer, to simulate real-time runs of the models rather than running models live as participants carried devices. In each model, the sole time-varying input was an indicator of environmental exposures along the past 5 h of GPS track. (See the “Methods” section for details on our environmental-exposure measure). The models also included person-level inputs reflecting demographics and SUD history. The output was a prediction of the probability of either heroin craving, cocaine craving, or stress (all reported in randomly prompted EMA entries on a smartphone) at any point in the next 90 min.

We should note, for readers unfamiliar with OUD and its treatment, that maintenance on methadone or buprenorphine reliably decreases illicit drug use and craving,^12–14^ but often does not eliminate them.^15,16^ Thus, it is both possible and clinically important to study craving in the context of those treatments, as we do here. We focused on craving for cocaine as well as illicit opioids because, of the nonopioid drugs commonly used by people with OUD during treatment, cocaine is especially common and problematic.^17,18^

In reporting our results, we emphasize not just overall accuracy, but also the components of accuracy: specificity, sensitivity, and—especially—positive predictive value (PPV) and negative predictive value (NPV). PPV and NPV indicate the trustworthiness of a prediction of presence or absence. This is the crux of how a JITAI will be experienced by users in real time: not sensitivity (“what percentage of my cravings will be detected?”) or specificity (*“*of the noncraving moments that constitute the bulk of my time, what percentage will be undisturbed by false alarms?”), but NPV (“does the app’s silence right now mean I’m not at risk of craving?”) and PPV (“is this craving alert necessary right now?”). For low-prevalence events, high specificity can mask very low PPV.^19^ Therefore, we began our analyses by characterizing the prevalence of our prediction targets.

## Results

### Prevalence per person

**Fig. 1:**
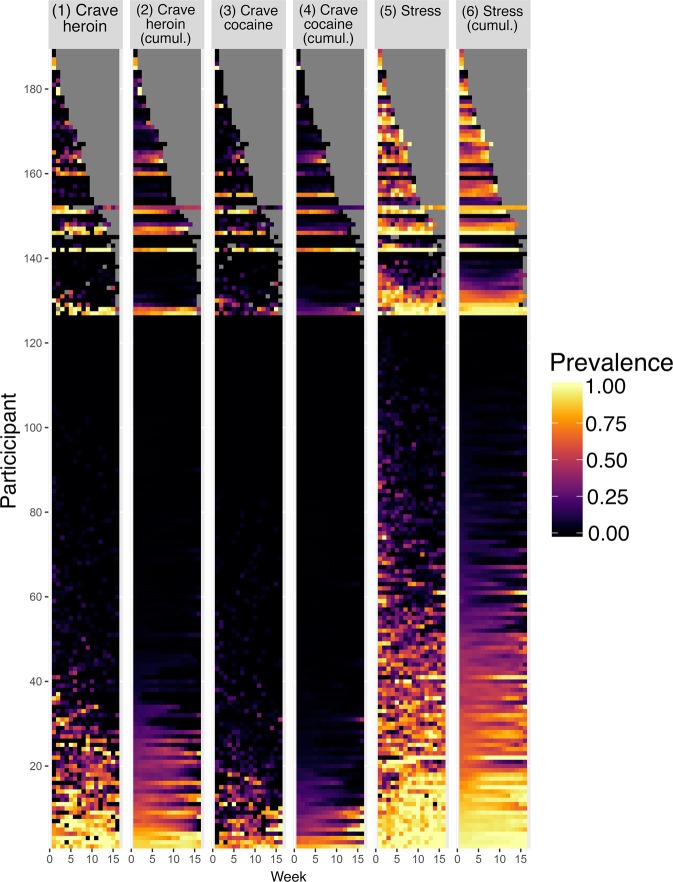
Prevalence of heroin craving, cocaine craving, and stress, for each participant. Data are shown as heat maps for the raw prevalences (columns 1, 3, and 5) and cumulative functions (columns 2, 4, and 6). Each of 189 participants is represented by one row on the *y*-axis, and each of 16 successive weeks (numbered 0–15) of 3x/day random-prompt entries is represented on the *x*-axis. The participants are sorted first by their duration in the study (gray indicates no data), then by their prevalence of the dependent variable. Cumulative functions smooth the data by showing a running tally of occurrences of the target variable up to that time point.

Figure 1 shows each participant’s prevalence for each of the three dependent variables: heroin craving, cocaine craving, and stress. In general, prevalences were low; many participants reported no occurrences. Columns 1, 3, and 5 show the data in raw form; columns 2, 4, and 6 smooth the data by using a cumulative function—i.e., any occurrence of the variable up to that time point. Raw prevalence showed substantial variability both between participants and over time. These fluctuations in prevalence complicate the use of traditional accuracy metrics, most of which are affected by prevalence. The use of a cumulative function helps stabilize between-week variability with each participant. Therefore, we used the cumulative-function data for the test of model accuracy that follow.

### Accuracy for each person by week, overall

**Fig. 2:**
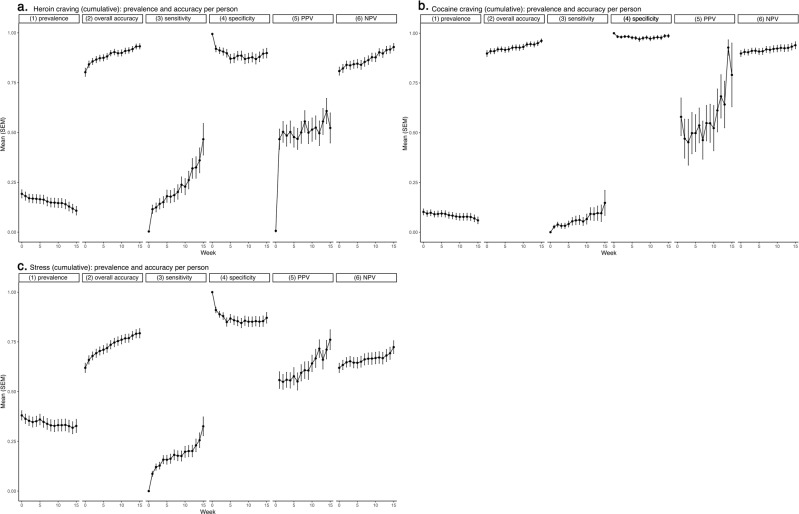
Prediction accuracy (and prediction-target prevalence) per person per week, across all participants. **a** Heroin craving, **b** cocaine craving, and **c** stress. For some measures, not all participants could be included: PPV could not be calculated when there was no prediction of presence, and sensitivity could not be calculated when the target event did not occur. Within each panel, 6 lines of data show the following. (1) A summary of the cumulative-prevalence data from Fig. 1, *N* = 187. (2) Overall accuracy, *N* = 187. (3) Sensitivity, *N* = 160 (heroin), 130 (cocaine), 181 (stress). (4) Specificity, *N* = 186 (heroin), 186 (cocaine), 184 (stress). (5) Positive predictive value (PPV), *N* = 68 (heroin), 149 (cocaine), 118 (stress). (6) Negative predictive value (NPV), *N* = 187. The accuracy statistics in lines 2–6, though shown as time series, do not literally display weekly accuracy for any one participant moving through time. Each time point represents a separate scenario. Run 0 shows accuracy when the model is run “off the shelf” for each participant, using other participants’ data to predict *all 16 weeks of responses* (in 90-min segments) for that participant. Run 1 shows accuracy when the model additionally includes one week of data from the target participant, predicting his or her *final 15 weeks of responses* (in 90-min segments). Run 2 shows accuracy when the model includes *two* weeks of data from the target participant, predicting his or her *final 14 weeks of responses* (in 90-min segments)—and so on.

Figure 2a–c shows accuracy (along with prevalence) by week, averaged across all participants.

For heroin craving (Fig. 2a), mean weekly prevalence started at 0.19 and decreased to ~0.11. Mean overall prediction accuracy started at 0.80 and increased to 0.93—but, as is common for low-prevalence prediction targets, the high overall accuracy was driven mostly by the relative ease of correctly predicting absence. This was reflected in high specificity (starting at 0.99, decreasing to 0.90) and high NPV, starting at 0.81, increasing to 0.93. The more challenging task of correctly predicting presence is reflected by sensitivity, which started at 0.00 in the “off the shelf” model at week 0, increased to 0.12 at week 1 with the inclusion of some individual tailoring data, and required all 16 weeks to become even as high as 0.47. Like sensitivity, mean PPV was 0.00 without tailoring, but unlike sensitivity, it quickly increased, jumping to 0.47 with just one week of tailoring, and reaching 0.56 at week 8.

For cocaine craving (Fig. 2b), mean weekly prevalence was even lower than that of heroin craving, starting at 0.10 and decreasing to 0.06. The accuracy results, accordingly, follow the same pattern as those for prediction of heroin craving, with an even greater difference between specificity (almost perfect) and sensitivity (never higher than 0.15). PPV did reach 0.93 and 0.79 for the final two weeks of prediction.

For stress (Fig. 2c), mean weekly prevalence was higher than that of craving; it started at 0.38 and decreased only slightly, to 0.33. This higher prevalence reduced the accuracy-inflating influence of high specificity: specificity started at 1.00 and quickly decreased to 0.87; overall accuracy started at 0.62 and increased to 0.80. Sensitivity started at 0.00, quickly increased to 0.09, and reached 0.33. PPV (after being undefined for the first week because there were no predictions of presence) started at 0.56 and slowly increased to 0.76.

For all three measures, the aggregated statistics in Fig. 2 suggest: (1) a non-tailored, “off the shelf” model (the model used at week 0) did not predict craving or stress; (2) as is typical for low-frequency prediction targets, overall accuracy was inflated by the influence of high specificity; (3) even so, mean PPV approached acceptable levels after just 1–8 weeks of tailoring.

**Fig. 3:**
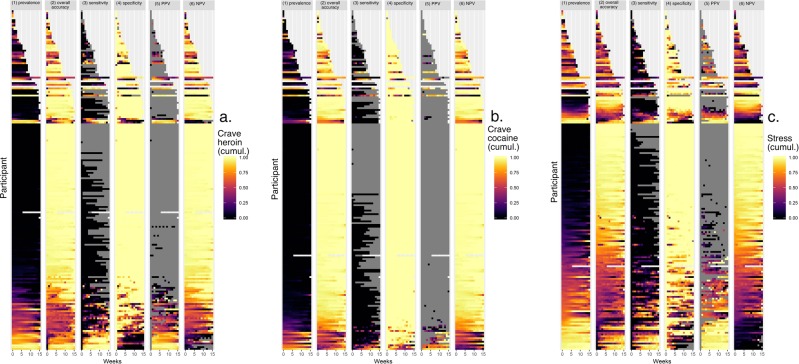
Prediction accuracy (and prediction-target prevalence) per person per week, for each participant. These heat maps recapitulate prevalence (column 1) and show model accuracy per person per week (column 2, overall accuracy; 3, sensitivity; 4, specificity; 5, positive predictive value; 6, negative predictive value). In columns 2–6, the color scheme is as follows. White: missing data. Gray: accuracy not calculable due to non-occurrence of event. Purple/black: low accuracy: Orange and yellow: high accuracy.

Figure 3a–c shows the accuracy (along with prevalence) data from Fig. 2a–c, person by person. For each of three prediction targets, the pattern was similar: accuracy was higher (closer to yellow) when participants were near the extremes, with either very low prevalence (driving up specificity and NPV) or very high prevalence (driving up sensitivity and PPV). For participants in middle ranges of prevalence, accuracy was lower (closer to purple or black).

Also clear in the figure was that PPV was often not calculable for a given participant in a given week, because the model that week included no predictions of presence for the rest of the participant’s data. Similarly, sensitivity was sometimes not calculable because the target event did not occur. Thus, even though it is important to consider accuracy at the levels of individuals and moments, the metrics used to express accuracy do not always lend themselves to the purpose. In the analyses we report in the next sections, on group differences in accuracy, we return to the aggregated level.

### Accuracy for each person by week, by sex

Although our aim was to develop individualized, temporally specific models, we also wanted to ensure that the models would pass basic checks for differential performance across demographic categories such as sex and race. We have previously published group-level findings, some from a cohort overlapping the current cohort, that showed small but detectable differences in drug-use behaviors as a function of sex^20,21^ or race^22^). Differences in prediction accuracy by sex or race, however, would be a sign that our approach to prediction had unforeseen pitfalls.

**Fig. 4:**
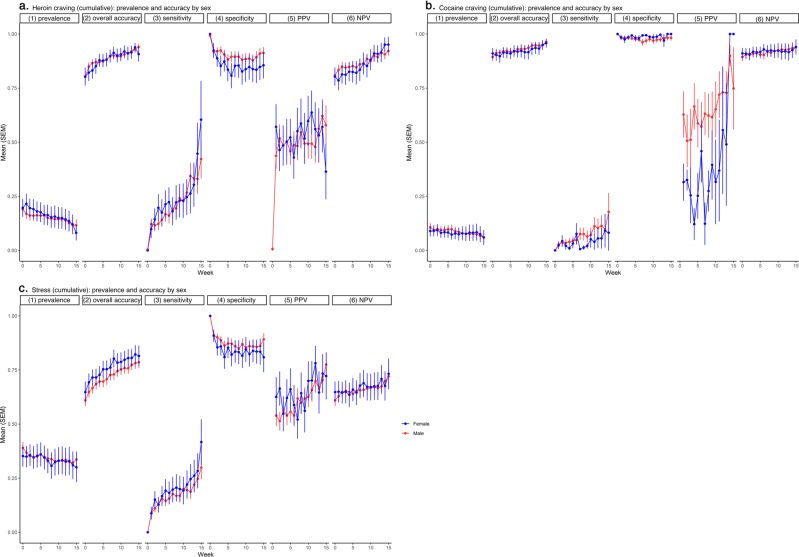
Prediction accuracy (and prediction-target prevalence) per person per week, by sex. **a** Heroin craving, **b** cocaine craving, and **c** stress. There were 141 men and 48 women. Details as in Fig. 2.

Figure 4a–c shows mean accuracy (along with prevalence) by week for women and men. There were no appreciable sex differences in prevalence or in any of the accuracy measures for heroin craving, cocaine craving, or stress. The median Cohen *d* value was 0.075, with a range from 0.02 (95% CL −0.45, 0.53) (heroin-craving PPV) to 0.18 (95% CL −0.11, 0.47) (heroin-craving specificity).

### Accuracy for each person by week, by race

**Fig. 5:**
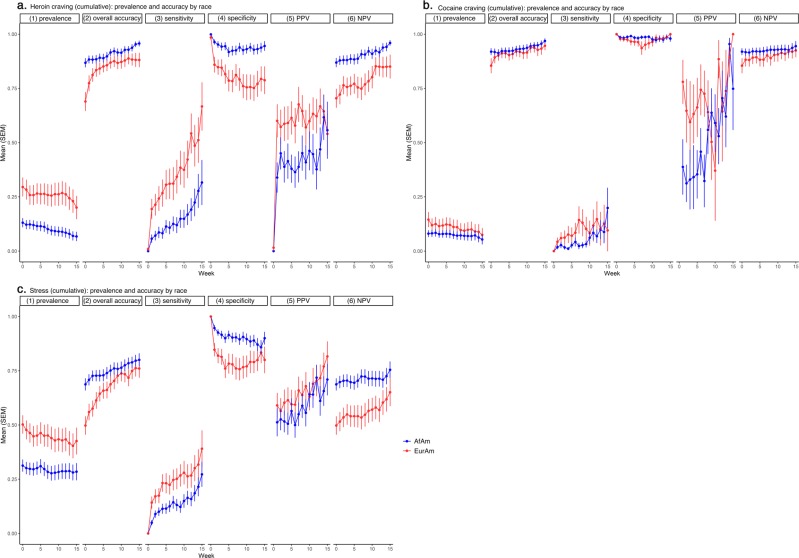
Prediction accuracy (and prediction-target prevalence) per person per week, by race. **a** Heroin craving, **b** cocaine craving, and **c** stress. There were 120 African-American (AfAm) participants and 66 European-American (EurAm) participants. Details as in Fig. 2.

Figure 5a–c shows mean accuracy (along with prevalence) by week for the two race categories in the sample: African Americans and European Americans. African Americans reported lower rates of heroin craving (*d* = 0.56, 95% CL 0.27, 0.87) and stress (*d* = 0.46, 95% CL 0.16, 0.75), and tended to report lower rates of cocaine craving (*d* = 0.18, 95% CL −0.11, 0.47). Overall accuracy was higher in African Americans than European Americans for prediction of heroin craving and stress; this difference in overall accuracy was driven by greater specificity and NPV and was offset by lower sensitivity and PPV.

### Accuracy for each person by week: differences among prevalence clusters

**Fig. 6:**
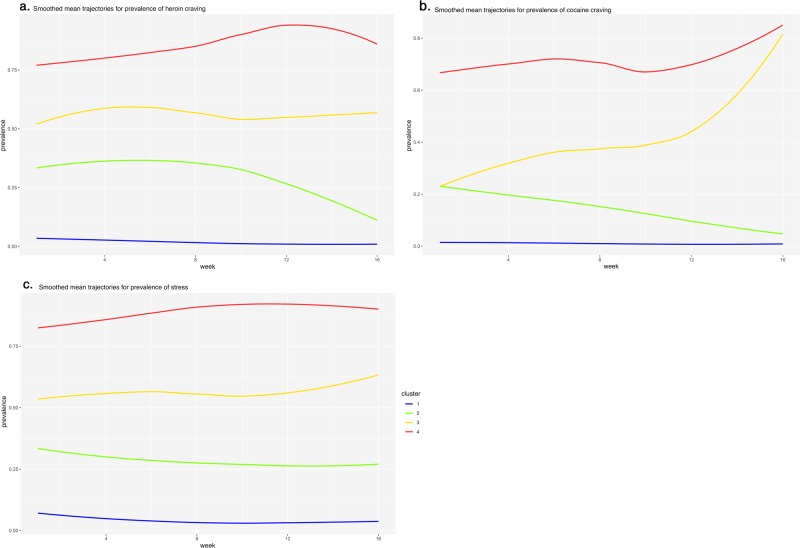
Smoothed mean trajectories of heroin craving, cocaine craving, and stress in empirically derived clusters of participants. We labeled the clusters (1) low prevalence, (2) medium-decreasing prevalence, (3) medium-increasing prevalence, and (4) high prevalence.

**Fig. 7:**
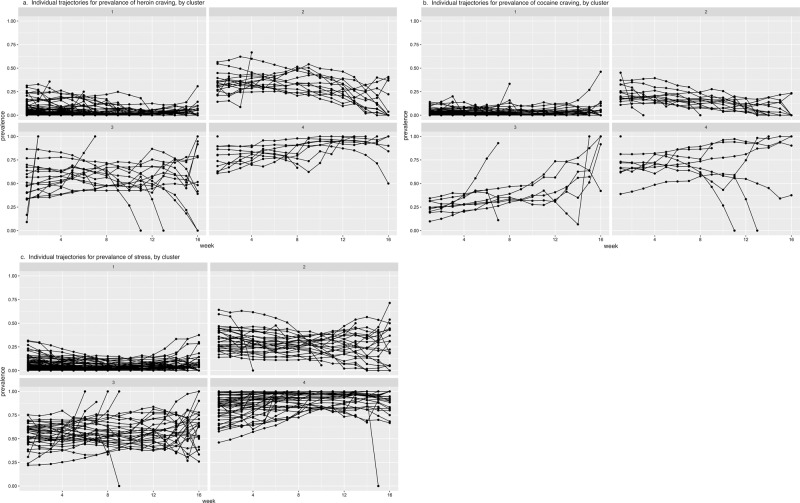
Individual participants’ trajectories of prevalence for heroin craving, cocaine craving, and stress, by cluster. The clusters are the same as those in Fig. 6.

Figures 6a–c and 7a–c show the empirically derived prevalence clusters for heroin craving, cocaine craving, and stress. We labeled the clusters (1) low prevalence, (2) medium-decreasing prevalence, (3) medium-increasing prevalence, and (4) high prevalence.

**Fig. 8:**
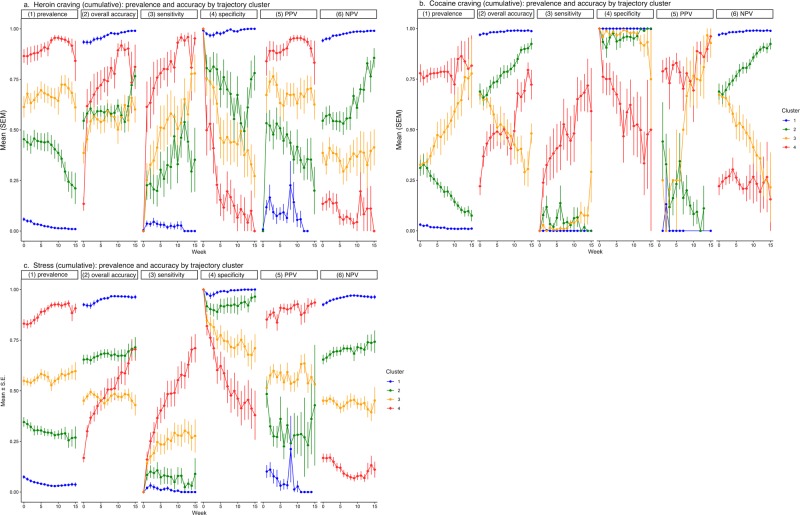
Prediction accuracy (and prediction-target prevalence) per person per week, by cluster. **a** Heroin craving, **b** cocaine craving, and **c** stress. Details as in Fig. 2. For measures of accuracy, the main effect of cluster is always significant, and, in Tukey pairwise comparisons, nearly all pairs of clusters differ from each other.

Figure 8a–c shows mean accuracy (and, again, prevalence) by week for the four trajectory clusters. As prevalence increased, overall accuracy for each of the three prediction targets decreased. Like the difference across racial groups, this greater overall accuracy at low prevalence was driven by greater specificity and NPV and was offset by lower sensitivity and PPV.

Differences by prevalence cluster appeared larger than differences by race. For example, the linear decrease in PPV for heroin-craving prediction across clusters (4 > 3 > 2 > 1) had a Cohen *d* of 3.50 (95% CL 2.64, 4.63). The corresponding effect sizes for other PPVs and NPVs ranged from 1.26 (95% CL 0.88, 1.65) (cocaine PPV: 4 > 3 > 2 > 1) to 4.15 (95% CL 3.51, 4.84) (stress NPV: 1 > 2 > 3 > 4).

In 15 additional multilevel models (one for each accuracy measure), we tested race and prevalence cluster simultaneously as predictors of accuracy. In every model, simultaneous inclusion of the two predictors greatly reduced the main effect of race (usually to less than half its original size, always with loss of its statistical significance) but not the main effect of prevalence cluster (which always remained statistically significant). Thus, the initial appearance of greater prediction accuracy for African Americans than for European Americans seems to be best viewed as an artifact of differences in the prevalence of the prediction targets.

## Discussion

Using only one form of continuous passive input (along with person-level demographic data), our machine-learning models predicted the occurrence of drug craving or stress 90 min into the future with excellent overall accuracy—as high as 0.93 by the end of 16 weeks—but this high overall accuracy was driven mostly by correct predictions of absence. For any given prediction of presence, “believability” (expressed as PPV) was lower, usually peaking in the high 0.70s toward the end of the 16 weeks. A PPV in the high 0.70s is often taken to be a successful result in the realm of JITAI development, but we have to temper our conclusions by noting that this was only our average PPV; it was lower when participants experienced the target event less frequently.

Our findings complement a body of published work by other investigators who have used machine learning with GPS-based, EMA-based, or sensor-based inputs to predict drug use,^23^ smoking,^24,25^ exercising,^26^ diet-related behaviors,^27–30^ and mood changes,^31–35^ on time scales ranging from hours to days. A closely related body of work used similar inputs for automated detection of current (not future) cigarette cravings,^36^ food cravings,^37^ stress,^38–41^ drinking,^42^ manic episodes,^43,44^ and mood.^45–50^ Prediction or detection accuracy in these studies was greatest for targets that had clear, enduring signatures, such as the transition from a depressive state to a manic state, which, with digital phenotyping, was detected with sensitivity and PPV of 0.97 on a whole-day time frame (that is, detection was counted as correct if the model flagged a transition before a whole day had elapsed).^44^ More elusive, however, was the detection of mental states such as stress, for which sensitivity and PPV were often below 0.50,^41,50^ and the prediction of future states or events, for which sensitivity, specificity, and PPV (when PPV was reported) tended to be in the 0.70s at best, occasionally reaching the 0.80s.^23,25,27,30,35,40^ These are mostly averages across all participants in a given study; many of the published reports do not provide information on how accuracy varied across people or time. In pointing that out, we do not mean to diminish what was accomplished in the cited studies; most of them are impressive demonstrations of at least partial success in a challenging area. Our own results should be viewed in that context.

We closely examined how the accuracy of our models differed from person to person—and we checked for differences among groups of people. Our goals here can be expressed in terms of the difference between precision medicine and personalized medicine. The term *precision medicine* has largely superseded the term *personalized medicine*; the change was intended to clarify that treatment matching would occur at the level of whole classes of patients, e.g., patients with some specific allele.^51^ But *personalized* medicine—in which randomization, statistical assessment of outcome, and treatment selection occur literally in one person at a time^52–54^—is very much the realm in which JITAI validation should occur. When we tested for difference in prediction accuracy as a function of sex and race, we were not attempting a crude approximation of precision medicine; we were checking for possible weaknesses in our method for personalized medicine.

Nonetheless, group-level indices might have a place in the practical deployment of a JITAI app. In the models we tested here, off-the-shelf accuracy for each new participant was exceedingly low. Some group-level information about a new participant (other than the group-level information we used) could be integrated into the first few weeks of model runs to provide a computational “warm start”. Based on our current findings, we are examining ways to determine each new participant’s trajectory cluster (low prevalence, high prevalence, etc.) as early as possible, thereby, we hope, making our momentary predictions more accurate more quickly. For people in different prevalence clusters, emphasis needs to be on different aspects of accuracy: high prevalence is a challenge to NPV and specificity; low prevalence is a challenge to PPV and sensitivity. JITAI developers should focus on the indices that are most difficult to achieve at their observed level of target prevalence.

One limitation of our method—that GPS track data were the only time-varying input to our models—is a limitation we imposed on ourselves purposefully. GPS logging imposes no burden of effort on participants and may raise fewer privacy concerns than digital phenotyping (because digital phenotyping entails logging of a broad range of information, usually including GPS). It is not surprising, however, that GPS tracks alone were insufficient for our predictive purposes; if anything, it is surprising that they worked as well as they did. Our next steps will include developing models that include some effortful input in the form of prior EMA entries; we will attempt to determine how dense or sparse this input must be to improve prediction accuracy. We are also planning to test some digital phenotyping and physiological monitoring.

Another limitation of the methods we reported here is that we did not systematically compare different types of machine learning (such as support vector machines or neural nets). In preliminary work with a similar data set, we found that randomForest models tended to produce clearer spikes of predicted risk than support vector machines. This says nothing about their respective accuracies, but it suggests that randomForest output would be more actionable for a JITAI. Mostly, however, we chose to use randomForest because it ran well with minimal adjustment to its default settings. We see this as strongly justifiable, given that we ran thousands of individual models. We focused on examining the performance of a single model setup over time.

In doing so, we chose to predict mental events such as craving and stress, rather than actual instances of drug use. We made that choice because craving and stress were more prevalent than use. Our own EMA studies have shown that lapses to drug use during treatment are preceded, on a scale of hours, by increases in craving and by changes in mood.^55,56^ Stress is related to lapses more complexly, but is often associated with them.^57^ Accurate prediction of any of those—drug craving, mood changes, or stress—is likely to help prevent lapses and would also be of inherent clinical value.

Having just referred to “accurate prediction,” we should acknowledge that we do not know what qualifies as sufficiently accurate for use in a JITAI app. We cannot state, for example, the threshold at which PPV becomes so low (and the false-alarm rate so high) that a patient will stop using the JITAI app. Most likely, it will be a matter of user preference, addressable by letting patients easily adjust the frequency with which content is pushed.

Further complexifying the issue, we should note that the meaning of accuracy for momentary-level detection or prediction is more elusive than it might seem. As we said when we reported group-level results from our pilot study,^5^ mental events with detectable physiological concomitants and important health consequences may occur without subjective awareness;^58,59^ thus, while self-report is an important check on detection accuracy, a user “denial” might not always indicate a false alarm. Conversely, a user “confirmation” might not always indicate a correct detection, because algorithmically triggered feedback can make respondents override their own self-knowledge and simply trust the machine’s assessment (unless investigators take care not to phrase the feedback in leading ways).^60,61^ Thus, accuracy is difficult not only to achieve, but sometimes even to define, and testing it live in the field (rather than on archival data, as we did here) can actually complicate matters. Even so, live testing is an important next step.

Finally, it is important to recognize that detection/prediction accuracy for a JITAI is a separate issue from the creation of the content the JITAI should deliver. The matching of interventional content to momentary situations is an active area of theory development^62–64^ and requires specialized study designs.^65^ Our research group is pursuing these two aims—prediction accuracy and content development—on parallel tracks until we are confident that we can combine them. Accuracy alone is a daunting challenge, yet accuracy alone does not guarantee benefit to patients. A validated JITAI should change clinically relevant outcomes, either momentarily,^65^ in the long term,^2^ or both, and that change should be greater than the change induced via some placebo algorithm that triggers interventions at random.^60,61^ The results we report here represent progress on one of the several paths that may converge on that outcome.

## Methods

### Study participants and treatment setting

At enrollment, participants were seeking treatment for opioid-use disorder (OUD) at a treatment research clinic in Baltimore, MD. During screening, participants completed the Addiction Severity Index (ASI)^66^ and the Diagnostic Interview Schedule (DIS IV)^67^ and were given physical examinations and psychological testing. The main inclusion criteria were: age 18–75 years, physical dependence on opioids, and residence in Baltimore City or one of the surrounding counties. The main exclusion criteria were: history of any DSM-IV psychotic disorder or bipolar disorder; current untreated Major Depressive Disorder; current physical dependence on alcohol or sedative-hypnotics; cognitive impairment precluding informed consent or valid self-report; conditions that preclude urine collection; or medical conditions that would compromise research participation.

After enrollment, participants began outpatient maintenance on methadone or buprenorphine and weekly individual counseling at the research site. Medications were administered in the clinic five to seven times per week, with medications given to take at home on weekends and major holidays. Urine drug screens were conducted two or three times per week.

The study was approved by the NIDA Intramural Research Program’s Institutional Review Board; all participants gave written informed consent. The study was covered by a federal Certificate of Confidentiality, assuring that data could not be subpoenaed. The study registration number in ClinicalTrials.gov is NCT00787423. All analyses focus on data collected from 189 outpatients who attended between 2009 and 2016; their demographic data are shown in Table 1.

**Table 1:** Clinical and demographic characteristics.

Total *N*	189
Opioid-agonist maintenance treatment	
Methadone	81 (43%)
Buprenorphine	108 (57%)
Age mean (SD) Sex	41.5 (9.6)
Male	141 (76%)
Female	48 (25%)
Race	
African American	120 (63%)
European American	66 (35%)
Multiracial	2 (1%)
Asian	1 (1%)
Years of education: mean (SD)	12.1 (1.5)
Marital status	
Married	14%
Never married	62%
Separated/divorced/widowed	24%
Employment status	
Full-time	46%
Part-time	22%
Unemployed	26%
Retired/disability/controlled environment	6%
Days used in past 30 before enrollment	
Heroin: mean (SD)	19.3 (11.9)
Cocaine: mean (SD)	4.4 (8.4)
Opioids other than heroin: mean (SD)	8.3 (10.4)
Years of use	
Heroin: mean (SD)	14.1 (10.2)
Cocaine: mean (SD)	5.6 (7.8)
Opioids other than heroin: mean (SD)	1.5 (2.6)
Route of administration	
Heroin (*n* = 175)	
Intranasal	61%
Intravenous	39%
Cocaine (*n* = 136)	
Smoking	54%
Intranasal	24%
Intravenous	22%
Opioids other than heroin (*n* = 144)	
Oral	92%
Intranasal	7%
Smoking	1%

### EMA data collection

After the first week of treatment, participants received a smartphone programmed to emit three audible prompts per day at random times during the participant’s typical waking hours. In each randomly prompted entry, participants rated their current heroin craving, cocaine craving, and stress. (Participants were also asked to initiate entries when they used drugs or experienced stressful events,^68^ and prompted to make an entry at the end of each day,^69^ but those data were not used in the present analyses). Participants carried the smartphones for up to 16 weeks and were paid $10–30 each week for completing at least 23 out of 28 weekly prompts. After two consecutive weeks of not meeting completion criteria, participants were not allowed to continue the study and were assisted with transfer into community-based addiction treatment.

The analyses presented here focus on responses to randomly prompted ratings of heroin craving, cocaine craving, and stress, each of which was rated 1–5 (anchored “not at all” to “extremely”) by participants. Because the data consisted mostly of “not at all” responses, forming an L-shaped distribution,^70^ we dichotomized them: ratings of 1 were recoded as 0; ratings of 2–5 were recoded as 1. In analyses to be reported elsewhere, we take other approaches to the problem of the L-shaped distribution, but dichotomizing none versus any is one reasonable approach when the goal is to make a dichotomous decision (specifically, to do nothing or to trigger a momentary alert/intervention).

### GPS data collection and pre-processing

During the EMA data collection period, participants carried small, no-display GPS loggers (BT-Q1000X, Qstarz International), which recorded geolocation every 20 m or every 15 min.^71^ The GPS loggers had internal Quality Assurance (QA) software that collects and computes information on the number of satellites, horizontal dilution of precision (HDOP), and position dilution of precision (PDOP). We identified and removed GPS points with signal error following methodology we have previously described.^5^ We processed the remaining GPS data to calculate speed and distance before and after each GPS point. We then used a speed filter (the R package argosFilter),^72^ which removes GPS points in each track if an adjacent point indicates an implausibly high velocity (≥ 31.3 m/s);^73^ the filter works iteratively until no such points remain. After running the speed filter, we recalculated values for speed and distance between GPS points.

### Tax-value data as a measure of environmental exposures

To operationalize environmental exposure, we used property-tax data from Maryland’s Department of Planning (http://planning.maryland.gov/Pages/OurProducts/PropertyMapProducts/Property-Map-Products.aspx), covering Baltimore City and County. In our prior GMA work, we had used an observer-rated measure of visible signs of disorder and poverty.^8^ This measure had been developed exclusively for use in cities; therefore, whenever a participant’s tracks left city limits, we had missing data. To obtain complete coverage and make all our track data usable, we switched to the use of tax-value data. We reasoned that property-category type and total estimated taxable value per parcel, taken together, would reflect socioeconomic conditions in a “wall to wall” fashion across the state. We mapped this in 30 m × 30 m pixels. The only type of land that has no taxable value in the database is roads and sidewalks; we assigned value to them by extending property values 50m into the street, using an inverse distance model (IDM).

### Linking EMA, GPS and tax-value data: preparation for machine learning

For each GPS point collected for each participant, we extracted the associated tax-value data and combined them into a single dataset. To obtain evenly spaced time points for training and validation of our machine-learning models, we aggregated the unevenly spaced GPS data into 30-min bins.^5^

For each EMA entry, we inserted the time stamp and participant identifier into the GPS/tax dataset, creating sequences of environmental-exposure information going back 24 h (1440 min) in 30-min bins. Missing GPS data from each 24-h sequence were replaced with the most recent prior data; when that was done, calculations of time and distance between GPS points were updated accordingly. When participants were outside Baltimore City and County, the exposure data were coded as missing. If this happened in an entire 30-min bin, the 30-min bin was coded as missing.

### Model development and testing

We developed our models with the intent to: (a) simulate real-time data collection as it would occur with a patient undergoing mobile treatment, and (b) test whether there was an optimal or necessary duration of data collection for each new patient before our machine-learning models could predict the patient’s cravings or stress accurately enough to give useful alerts.

We used the randomForest algorithm, a machine-learning method that aims for case-by-case accuracy rather than explanatory clarity.^11^ Each model was calibrated with 200 trees, and the number of variables randomly sampled at each split was set to one third of the total candidates.

Our models used geotagged tax-value data as the only time-varying predictor, simulating a zero-burden form of assessment. Five hours of those data went into each prediction; we chose that time frame because it showed the strongest relationships with current craving and stress in our pilot data.^5^ Person-level predictors in the models were: sex; age; race (African American or European American); Hispanic ethnicity; education (high-school graduate vs. not); years of education; marital status; professional training (any vs. none); current employment (any vs. none); days of using heroin, other opioids, or cocaine in the 30 days before enrollment; lifetime years of use of heroin, other opioids, or cocaine; and typical route of administration of each drug. Race and ethnicity were self-reported by participants.

The output of each model was the occurrence or nonoccurrence of the target event (heroin craving, cocaine craving, or stress) at any time in the next 90 min (i.e., a single prediction of *presence* if the model projected that the target event would occur in any of the next three 30-min bins, or a single prediction of absence if not).

We first tested accuracy for a model that was run as if “taken off the shelf” at week 0 for each participant, using other participants’ data to predict all 16 weeks of responses (in 90-min segments) for that participant. We used a “leave-one-out”^11^ cross-validation approach, with a training database of 188 participants used to make predictions for the held-out participant. We implemented this modeling scenario training on each of the 189 participants, resulting in 189 models trained and validated at Week 0.

We next tested whether model accuracy would increase with greater tailoring, week by week. Thus, our models for Week 1 assessed accuracy with the inclusion of one week of data from the target participant, predicting his or her final 15 weeks of responses (in 90-min segments). Our models for Week 2 assessed accuracy with the inclusion of *two* weeks of data from the target participant, predicting his or her final 14 weeks of responses (in 90-min segments)—and so on. We continued this through week 15, when we were left with only week 16 to predict. Thus, there were up to 16 model runs for each of the 189 participants.

### Accuracy statistics

We used the R package *epiR*^74^ to summarize prevalence of the prediction target and to calculate overall accuracy and its components (of which we report sensitivity, specificity, PPV, and NPV). We calculated model performance per person per week, first aggregated across the whole sample, then broken down by the basic demographic variables sex and race.

### Inferential statistics on person-level correlates of accuracy

To assess whether accuracy differed by sex or race, we used random-intercept multilevel models (SAS Proc Mixed) in which the predictors were sex (or race), week, and the interaction of the two, and the dependent variable was the accuracy measure (total accuracy, sensitivity, specificity, PPV, or NPV). Multilevel models accommodate incomplete repeated-measures data without requiring imputation of missing data points. The resultant F tests can be used to calculate effect sizes and confidence intervals.^75^

We used similar mixed models to test how prediction accuracy was affected by the prevalence of the prediction targets. To operationalize prevalence as a person-level predictor, we fit latent-class growth models (LCGM),^76^ clustering participants by their trajectories of week-by-week prevalence for heroin craving, cocaine craving, and stress. We fit those models with the R package lcmm,^77^ using the BIC and fit between predicted values and observed values to select the final LCGM. In each case, the best-fitting solution consisted of four clusters, which we named low prevalence, medium-decreasing prevalence, medium-increasing prevalence, high prevalence. We used cluster as a predictor in another set of multilevel models, again using the resultant F tests to calculate effect sizes and confidence intervals. Finally, because accuracy seemed to differ as a function of both race and prevalence cluster, we ran models in which we included both as predictors.

In all multilevel models, alpha was set at 0.05, two-tailed. Our main interest, however, was not on null-hypothesis significance tests, but on describing the extent and reliability of any person-level correlates of prediction accuracy. Therefore, we report the results of these models only as brief summaries of the resultant effect sizes and confidence intervals.

### Reporting summary

Further information on research design is available in the Nature Research Reporting Summary linked to this article.

## Supplementary information

Reporting Summary

## Data Availability

Because these data—individual GPS tracks along with self-reports from people in treatment for opioid-use disorder and cocaine-use disorder—are both sensitive and difficult to anonymize, they are not being placed in a publicly accessible repository. We can make data available to other investigators in accordance with the terms on which our participants agreed to provide those data. Our IRB-approved consent form tells participants: “Research partners outside the NIH sign an agreement with the NIH to share data. This agreement indicates the type of data that can be shared and what can be done with that data. This partnership must be approved [by our IRB] before your data can be shared”.
